# Non-RhD alloimmunization in pregnancy: an updated review

**DOI:** 10.61622/rbgo/2024AO22

**Published:** 2024-03-15

**Authors:** Sabrina Menes Ares, Luciano Marcondes Machado Nardozza, Edward Araujo Júnior, Eduardo Félix Martins Santana

**Affiliations:** 1 Albert Einstein School of Medicine Department of Maternal and Child São Paulo SP Brazil Department of Maternal and Child, Albert Einstein School of Medicine, São Paulo, SP, Brazil.; 2 Federal University of São Paulo Paulista School of Medicine Department of Obstetrics São Paulo SP Brazil Department of Obstetrics, Paulista School of Medicine, Federal University of São Paulo, São Paulo, SP, Brazil.; 3 Municipal University of São Caetano do Sul Medical course São Caetano do Sul SP Brazil Medical course, Municipal University of São Caetano do Sul, São Caetano do Sul, SP, Brazil.

**Keywords:** Non-Rh alloimmunization, Erythroblastosis, fetal, Prevalence, Fetal diseases, Rh Isoimmunization, Blood group antigens, Pregnancy

## Abstract

RhD alloimmunization in pregnancy is still the main cause of hemolytic disease of the fetus and neonate (HDFN). Nevertheless, there are other antigens that may be associated with the occurrence of this phenomenon and that have been growing in proportion, given that current prevention strategies focus only on anti-RhD antibodies. Although not widespread, the screening and diagnostic management of the disease caused by these antibodies has recommendations in the literature. For this reason, the following review was carried out with the objective of listing the main red blood cell antigen groups described — such as Rh, ABO, Kell, MNS, Duffy, Kidd, among others — addressing the clinical importance of each one, prevalence in different countries, and recommended management when detecting such antibodies during pregnancy.

## Introduction

Alloimmunization is an immune process in which antibody production occurs upon exposure to non-self-antigens. In the gestational context, previous maternal exposure to certain alloantigens can lead to the formation of immunoglobulins that, if they pass the placental barrier, harm the fetus.^(1)^ This phenomenon is well described in relation to the formation of alloantibodies against red cell antigens. Namely, the pregnant woman, previously exposed to red cells — either by maternal-fetal bleeding, transfusions, sharing needles, or other forms of blood exposure^(2)^ — produces antibodies that bind to the red blood cells of the fetus or neonate causing hemolysis. When clinically significant, the condition is called hemolytic disease of the fetus and neonate (HDFN). In severe cases, the disease can progress to fetal hydrops, which is characterized by severe anemia, hepatic and splenic hematopoiesis, heart failure, and edema.

Among all the erythrocyte alloantigens, the one with the greatest clinical significance and the greatest number of publications is anti-Rhesus (Rh) D.^(1)^ However, amid the 345 red blood cells antigens listed by the International Society of Blood Transfusion, more than 50 may be associated with the occurrence of HDFN.^(3)^ Among them are other antibodies against the Rh system in addition to RhD, and also against other blood group systems such as ABO, Kell, Duffy, Kidd, MNS, and others.^(4)^

Since RhD alloimmunization is the most relevant, prevention methods are almost exclusively aimed at it. Consequently, as a result of the dissemination of these strategies, the incidence of HDFN due to anti-RhD alloantibodies has decreased significantly. Although the prevalence of anti-RhD antibodies in screening tests varies according to population, there is no denying that the administration of anti-D immunoglobulin has had a positive impact. In Western countries, for example, the incidence of RhD alloimmunization has fallen from 16 to 0.3%.^(5)^

Meanwhile, the occurrence of fetal or neonatal harm from other alloantibodies has increased in proportion, for most of which there are still no established screening protocols during pregnancy or testing by blood transfusions. The current incidence of alloimmunization due to non-RhD antibodies, also in Western countries, is 0.28-0.33%.^(6–8)^ Thus, the clinical importance of non-RhD alloimmunization and the need to address this topic is increasing.

Given the clinical relevance of the topic, this updated review was conducted in order to evaluate the main non-RhD alloantibodies with clinical significance, as well as their prevalence and effects. Likewise, the screening and management strategies facing the occurrence of alloimmunization for these alloantibodies were reviewed.

## Clinical significance

Once maternal alloantibodies against erythrocyte antigens are present, the occurrence of alloimmunization depends on some factors, such as the class of immunoglobulin, its specificity, the antigenic volume expressed by the fetus or neonate, and the maternal blood antibody concentration.^(5)^

To cause injury, immunoglobulins must pass through the placental barrier, that is, they must belong to the IgG class. Consequently, antibodies expressing only IgM, such as Lewis I and P1, are not able to cause disease.^(5,9)^ Similarly, antibodies against Lutheran and Yt groups, because they are poorly expressed by fetal cells, virtually do not cause alloimmunization.^(5)^ In addition, there are some antibodies against the Cromer group that, although belonging to the IgG class, bind to a protein called complement decay accelerating factor, preventing its placental passage.^(10)^ Therefore, there are erythrocyte antigens that, despite being associated with alloantibody production, are not associated with unfavorable clinical outcomes, since they do not come into contact with fetal cells.

However, there are other blood groups whose respective antibodies are related to the occurrence of disease. Rh and Kell groups are usually more associated with the development of severe disease, with a high-risk of occurrence. Meanwhile, ABO, Duffy, Kidd, MNS, Diego, and other less commonly mentioned groups have a lower risk and are generally associated with a lower incidence of severe cases.^(4,5,11)^ It is worth remembering that the risks and clinical importance also vary according to the different antibodies within each group, with anti-Rhc, anti-RhE and anti-K alloantibodies being the most noteworthy.^(3,4)^

Furthermore, in addition to the isolated effect of the aforementioned antibodies, they can occur in association with anti-RhD or with each other, and thus cause more severe outcomes. The compound antibodies related to more severe disease are anti-CD, anti-cE and other anti-Rh antibodies in conjunction with anti-RhD.^(8)^ Therefore, the other immunoglobulins against the Rh system may not only cause HDFN, but aggravate cases of anti-RhD alloimmunization.

## Prevalence

Besides the fact that not all respective antibodies have clinical significance, it is important to consider that their prevalence varies according to each population.

### Western countries

In Western countries, Rh and Kell blood groups should be highlighted, as analyzed in studies conducted in the United States of America, Ireland, the Netherlands and Canada. An analysis conducted in New York with data from 1993 to 1995 revealed, among 37506 blood samples, 452 women with positive screening for red blood cells alloantibodies, being, in addition to anti-RhD, the most frequent: anti-Kell (22%), anti-RhE (14%), anti-Rhc (5.8%), anti-Fya (5.4%), anti-RhC (4.7%), anti-MNS (4.7%) and anti-Jka (1.5%).^(11)^ In comparison with other past studies that evaluated the population of Minnesota, New York, Australia and Sweden, the authors noticed a major increase in the frequency of antibodies against the Kell group.^(11)^ In Ireland, 34913 samples were studied from 1999 to 2000, among which 186 women showed clinically significant non-RhD antibodies. Among them, the most prevalent were anti-RhC (26.3%), anti-Kell (22.0%) and anti-Rhc (12.3%).^(6)^ In a prospective cohort conducted in the Netherlands, 305000 pregnancies in the years 2002 to 2004 were included, among which 1002 had non-RhD alloantibodies. The most common ones found were anti-RhE (28.8%), anti-K (21.1%) and anti-Rhc (15.1%).^(7)^ Although not a western country, similar results were observed in a retrospective study in Israel between January and December 2011, in which 900 of 90948 women presented with non-RhD alloantibodies, with the same ones mentioned above being most common, with respective frequencies of 22.7%, 16.1% and 10.9%.^(12)^ Another retrospective study, conducted in Canada, surveyed data from 2006 to 2010. Of 155153 pregnancies evaluated, 559 had positive screening for red cells alloantibodies, the most frequent, excluding anti-RhD, being anti-RhE (48.4%), anti-Rhc (15.7%) and anti-Jka (10.3%).^(13)^ In Brazil, a cohort was conducted in between 2017 and 2018, evaluating 2391 pregnant women, of whom 60 had positive antibody screening. Non-RhD antibodies represented 47.7% of the sample. The antibodies found were anti-C (15.5%), anti-Lea (11.1%), anti-Dia (6.7%), anti-E (6.7%), anti-Leb (6.7%), anti-M (6.7%), anti-K (4.4%), anti-Fya (4.4%), anti-Cw (2.2%), anti-Fyb (2.2%), and anti-Jka (2.2%).^(14)^

### Eastern countries

Compared to Western countries, Eastern populations have a lower prevalence of anti-RhD alloantibodies, although the severity of the HDFN caused by these is higher.^(15)^ As in the West, other antibodies against the Rh group are also found, but there is a special emphasis on the MNS group. In a study conducted in Taiwan, 23886 data were gathered from 1991 to 2000, with 15 cases of HDFN. Among the associated antibodies were: anti-RhE (40%), anti-RhE in association with anti-Rhc (20%), anti-RhD (20%), anti-Mi (13.3%) and anti-RhC (6.6%).^(15)^ Whereas in a cohort conducted in China between 2005 and 2019, besides anti-RhD alloantibody, anti-M was the most common found alone. Anti-RhEc and anti-RhCe were the most frequent found in association with anti-RhD.^(16)^ In India, a prospective study from 2013 to 2015 obtained a sample of 2336 patients, of whom 3.68% screened positive for antibodies. The most frequent ones found, besides anti-RhD alone were: anti-Leb (12%), anti-H (Bombay phenotype) (7%), anti-RhD in association with anti-RhC (5%), anti-RhG (5%), anti-Rhc (5%), anti-RhE (2%), anti-Rhe (2%), anti-M (2%), anti-Lea (2%).^(17)^ Meanwhile, a cohort conducted in Pakistan published in 2014 analyzed 1000 pregnant women, of whom 1.6% had non-RhD antibodies, the most frequent being anti-M (15%), anti-Lea (15%), anti-RhC (5%), anti-Rhe (5%), anti-Leb (5%).^(18)^

## Blood groups

### Rhesus

The Rh blood group has the greatest immunogenic capacity compared to the others. There are more than 49 antigens described, the main ones with clinical significance being: D, C, c, and E.^(19)^ The group is derived from the RHD and RHCE genes. The former encodes the D antigen protein and the latter can lead to the CE, Ce, cE and ce phenotypes,^(20)^ forming eight different possible gene complexes listed in order of prevalence in white individuals: CDe, cde, cDE, cDe, Cde, cdE, CDE and CdE.^(21)^ Following anti-RhD, anti-Rhc is the Rhesus group antibody associated with the greatest severity. It can cause alloimmunization and has a similar hemolytic effect as anti-RhD.^(3)^ Although 20 times less immunogenic than anti-RhD, anti-Rhc antibody can induce severe hemolytic anemia.^(22)^ However, HDFN, fetal death and morbidity from this antibody are rare outcomes.^(23,24)^ Anti-RhC, anti-RhE, and anti-Rhe, on the other hand, occur in lower titers and, if associated with the presence of anti-RhD, exert an additive hemolytic effect, potentiating the severity and magnitude of red cell injury.^(3)^ Among them, anti-RhE succeeds anti-Rhc in clinical importance, being associated with lower disease risk, with generally mild severity.^(3)^

### ABO

Although it does not cause severe alloimmunization, the ABO group has the most immunogenic antigens. They are: A, B, AB, and A1. The group is derived from the ABO gene, which has the codominant A and B alleles and the recessive O, giving rise to the phenotypes A (A1 and A2), B, AB, and O.^(20)^ Antibodies are naturally produced against any ABO antigen that is not expressed by the individual’s red cells, without the need for transfusion or prior gestational exposure. This occurs when the immune system is exposed to saccharides present in foods and microorganisms that are very similar or equal to the aforementioned antigens. Therefore, these antibodies are universally produced except by individuals with an AB phenotype.^(20)^ ABO blood incompatibility is the most common, affecting 15 to 25% of pregnancies^(25)^ and usually occurs in type O pregnant women whose fetus has blood type A, B, or AB. This is due to the fact that the antibodies produced by O individuals are of the IgG class, while those produced by A or B individuals are more commonly of the IgM class.^(20)^ Despite the universality of these antibodies, only 1% of individuals with incompatibility develop alloimmunization, which is rarely severe,^(25)^ since the fetal cells do not express the A and B antigens in large quantities.^(20)^ When it does occur, usually the only manifestation is jaundice, but in some cases there may be a need for phototherapy or transfusion. There are rare reports of fetal hydrops.^(26)^ It is believed that, in individuals of African descent, there is a higher risk of jaundice, due to the higher incidence of O-B incompatibility in this population, which is supposed to be more severe. However, there is no consensus that O-B mismatch leads to worse outcomes than O-A, as some studies point to this difference, while others show similarity between the two groups.^(27,28)^

### Kell

Kell is the third group with the most immunogenic antigens, following the ABO and Rh systems.^(20)^ It is derived from an extremely polymorphic gene and can encode 25 different antigens, such as K, k, Ko, Kpa, Kpb, Jsa, Jsb, and others.^(20,28)^ The K antigen is the most antigenic, while the others relate to a lesser extent to blood incompatibility. That is, the anti-K antibody is frequently associated with severe alloimmunization, while the others mentioned above can cause mild disease, and there are also other antibodies with no reports of clinical repercussions.^(9)^ Although most countries do not routinely perform typing for the Kell group in blood donations, blood transfusion is the main form of sensitization.^(29)^ There are other risk factors, such as multiparity associated with K-positive partners, or the association of previous pregnancies and transfusions.^(29,30)^ It is described that anti-K alloimmunization is associated with a number of negative outcomes for the fetus and neonate: need for intrauterine transfusion, hydrops, fetal death, and fetal anemia.^(3,31)^ Moreover, the manifestations can begin rapidly, at 18 to 20 weeks of gestation, and become severe at 20 to 25 weeks.^(32)^ This severity is not explained solely by the hemolytic action of anti-K antibodies. It is known that this antibody can suppress fetal erythropoiesis by destroying erythroid precursors in the bone marrow. The Kell protein, present in Kell positive individuals, is structurally similar to the neutral endopeptidases family (neprilysin zinc-metalloproteinase). That is, it may be associated with erythrocyte differentiation and growth, so that when destroyed, it compromises erythropoiesis.^(3,33)^ Since erythroid precursors do not contain hemoglobin, their destruction is not so much related to jaundice, but can cause severe anemia.^(20)^ Therefore, it is understood that antibodies against the Kell group lead to fetal anemia not only due to hemolysis, but also by fetal bone marrow depression, contributing to a greater severity of the condition and to fetal harm occurring even when the antibodies are present at low titers.

### Duffy

There are only two antigens of the Duffy group: Fya and Fyb, which are encoded by codominant alleles FY*A and FY*B, defining the following phenotypes: Fy(a+b-), Fy(a-b+), Fy(a+b+) and Fy(a-b-). These antigens are known for their role in the penetration of Plasmodium vivax and Plasmodium knowlesi merozoites into red blood cells, so that Fy(a-b-) erythrocytes, more common in Africans, are not invaded by these parasites.^(34)^

Duffy antigens are weak stimuli for antibody production.^(35)^ However, although uncommon, the main form of sensitization to Fya antigen is by blood transfusion and rarely arises from previous pregnancies, and anti-Fya antibodies are generally related to the occurrence of moderate alloimmunization.^(3)^ There are reports in the literature ranging from subclinical,^(36)^ to mild^(37,38)^ and severe HDFN.^(39,40)^ Fyb, on the other hand, is 20 times less common and its antibodies are not associated with the occurrence of alloimmunization.^(3,34)^

### MNS

The MNS blood group is composed of over 40 different antigens, with M, N, S, s and U being the most associated with alloimmunization.^(3)^ The group is derived from the GYPA and GYPB genes, which encode the M and N; S and s alleles respectively, while deletion of the GYPB gene leads to expression of the U antigen.^(20)^ Mutations can lead to the production of other antigens, such as Mia, Mta, Vw, Mur, Hil and Hut, which can also cause the disorder, but with less importance.^(9)^ Among the listed antigens, the ones most associated with alloimmunization are S and s, the latter being more frequent than the former. This is because anti-S and anti-s antibodies can cause severe hemolysis.^(20)^ The anti-M antibody, on the other hand, usually occurs in the IgM form and is therefore less related to the disease. However, in rare cases, it can be converted to IgG and thus has the potential to cause severe disease.^(3)^ Similarly, anti-N disease is also quite rare, but can cause mild hemolysis.^(41)^ U antigen is frequent in the population,^(3)^ while anti-U antibody is rare and occurs only in the African population, at a proportion of 1%.^(42)^ It can cause mild to severe alloimmunization, with reports in the literature of neonates developing late-onset anemia, requiring transfusion, and requiring intensive care unit care.^(3)^

### Kidd

The Jk1, Jk2 and Jk3 antigens make up the Kidd system. They are products of two codominant alleles (Jka and Jkb) of the SLC14A1 gene.^(22)^ The Jk null (a-b-) phenotype leads to the production of anti-Jk3 antibodies and, although very rare in most populations, can cause alloimmunization.^(3,20)^ Although there are reports of fatal disease from anti-Jk3, such severity is an uncommon outcome, with most cases, although rare, being mild.^(20)^ Similarly, anti-Jka and anti-Jkb antibodies against the Jk1 and Jk2 antigens respectively also cause mild HDFN.^(9)^

### Others

There are also other blood groups associated with alloimmunization, but at a lower incidence and clinical importance, given the low number of reported cases. Some examples are group P, which can cause severe disease when the anti-PP1Pk antibody is expressed.^(9)^ Or the Diego group, in the presence of the anti-Dia and anti-Dib antibodies, which are more common in the population with mongoloid ancestry, and can cause mild to severe alloimmunization.^(9)^ However, there is an endless list of blood groups with reports of HDFN induced by their respective antibodies, such as Colton, Dombrock, Gerbich, Scianna, Xg, Becker, Evans, Hunt, Wright, and others.^(3,9)^ Table 1 describes the main blood groups studied, their antigens and the respective risk and severity of HDFN caused by them.

**Table 1:** Main blood groups with their respective antibodies, and risk e severity of alloimmunization

Blood group	Main antibodies	Risk of alloimmunization	Severity of alloimmunization
Rh	RhD	High	Severe
	Rhc	High	Mild to severe
	RhC	High	Mild
	RhE	Medium	Mild
ABO	A, B e A1	Low	Mild
Kell	K	High	Severe
	k, Ko, Kp^a^, Kp^b^, Js^a^, Js^b^	Medium	Mild to severe
MNS	S and s	Low	Mild, potentially severe
	M	Low	Mild, potentially severe
	N	Low	Mild
	U	Low	Mild, potentially severe
Duffy	Fy^a^	Medium	Mild to severe
	Fy^b^	No risk	
Kidd	Jk^a^, Jk^b^, Jk3	Low	Mild
P	P1	No risk	
	PP1pk	Low	Mild to severe
Diego	Di^a^ e Di^b^	Low	Mild to severe
Lewis	Le^a^ e Le^b^	No risk	
I	I	No risk	
Lutheran	Lu^a^ e Lu^b^	Almost no risk	

## Management

### Screening

In most countries, prenatal screening is performed with ABO and Rh blood typing. However, there are other antibodies, not routinely screened, that can cause harm to the fetus. It is recommended that care in cases of presence of non-RhD antibodies be the same as in RhD alloimmunization, except in cases of Kell sensitization, in which titration values are less accurate and have less clinical correspondence.^(21)^ In other words, all pregnant women should have a history of alloimmunization investigated and should have ABO and Rh typing and testing for irregular antibodies in the first trimester, after delivery and in the presence of complications such as bleeding or trauma, for example.^(21)^ In cases of high-risk of alloimmunization according to the couple’s history, the pregnant woman should be referred to a center specialized in Fetal Medicine.^(1)^ In such cases, maternal antibody titrations are not useful and the fetus should be evaluated with serial middle cerebral artery Doppler from 16 to 18 gestational weeks, once the presence of fetal antigen is confirmed by amniocentesis or maternal plasma free fetal DNA testing.^(3)^ For patients with no history, if any antibody is detected, the risk of alloimmunization should be considered by evaluating the type of antibody and its ability to cause disease.^(43)^ If there is a risk, the titration of the antibody in maternal blood should be determined. Titers higher than 1:8 to 1:32 (depending on the service) denote risk of developing disease and the need for monitoring fetal anemia.^(21)^ The risk of alloimmunization is related to whether or not this critical value is extrapolated, and the magnitude of titers is not related to degrees of disease. That is, higher titers do not necessarily translate into a more severe disease.

### Further investigation

In the presence of irregular antibodies at critical titers, further investigation is required, assessing whether the fetus produces the corresponding antigen.^(3)^ The fetal genotype should be evaluated via the maternal blood free fetal DNA test, which is able to detect the D, C, c, E, and K1 antigens with sensitivity and specificity of almost 100%.^(1,3)^ To investigate other antigens, amniocentesis with PCR is performed, with sensitivity of 98.7% and specificity of 100%, but always considering its risks.^(21)^ Chorionic villus sample should be avoided due to the high risk of complications.^(21)^ Thus, if the fetus is negative for the antigen in question, the investigation can be discontinued, as false-negative rates are only 1-3%,^(21)^ although in some cases it is recommended to obtain a second sample to exclude false-negatives.^(43)^ While, if positive, monitoring for complications is necessary.

### Monitoring

It is recommended to monitor antibody titers. If the initial titers are lower than 1:32, it is necessary to repeat the exam monthly until 28 weeks of gestation, and every two weeks after reaching this gestational age.^3^ In case of initial values equal to or greater than 1:32, the titration should be repeated every 15 days and complications such as hydrops and fetal anemia should be evaluated.^(43)^ Once the risk of fetal anemia is confirmed, follow-up with ultrasonography and Doppler of the middle cerebral artery should be performed, a technique that has increasingly replaced amniotic fluid analysis.^(44)^ Before 18 weeks of gestation, signs of fetal hydrops are investigated, which is defined as the pathological accumulation of fluid in two or more fetal compartments (pleural effusion, pericardial effusion, ascites, subcutaneous edema) and may be accompanied by polyhydramnios and placental edema.^(43)^ From 18 weeks, Doppler is used to assess the middle cerebral artery peak systolic velocity (MCA-PSV) every 1 to 2 weeks.^(3)^ In the presence of severe fetal anemia history, Kell alloimmunization, or very high titers, investigation can be initiated from 16 weeks gestational age.^(43)^ The MCA-PSV assessment is a noninvasive test that is considered the gold standard for screening fetal anemia,^(1)^ but should be performed only by trained professionals. The multiple of the median (MoM) is used, obtained from the ratio between the measured value of MCA-PSV and the median established for a given gestational age. Values greater than 1.5 MoM diagnose moderate to severe fetal anemia, with 100% sensitivity and 12% false-positive rate, and it is considered severe in those patients with measurements greater than 1.55 MoM.^(45)^ However, from 34 to 35 weeks, there is a higher proportion of false-positives.^(21)^ With measurements greater than 1.5 MoM, cordocentesis is indicated for confirmation of fetal anemia and intrauterine transfusion.^(3)^

### Kell alloimmunization

Specifically in cases of Kell alloimmunization, the management is somewhat divergent. Anti-Kell antibodies usually have less accurate titers and therefore critical titers are lower: indicating a need for monitoring when greater than 1:4.^(3,33)^ Since anemia in fetuses with Kell alloimmunization is not only due to fetal erythroblastosis but also to inhibition of erythroid precursors, amniotic fluid analysis may not be sufficient to detect it.^(44)^ Thus, the evaluation of MCA-PSV is even more important and can be started at 16 weeks of gestation.^(43)^ In addition, fetal anemia can worsen rapidly, which calls for more frequent evaluations.^(44)^ In contrast to the other antibodies, where the risk of severe anemia occurs only early in gestation, for patients with suspected Kell alloimmunization, repeat testing is indicated even after 28 weeks of gestation.^(1)^ The rest of the management recommendations resemble those regarding the other antibodies.

### Therapeutic management

**Figure 1:**
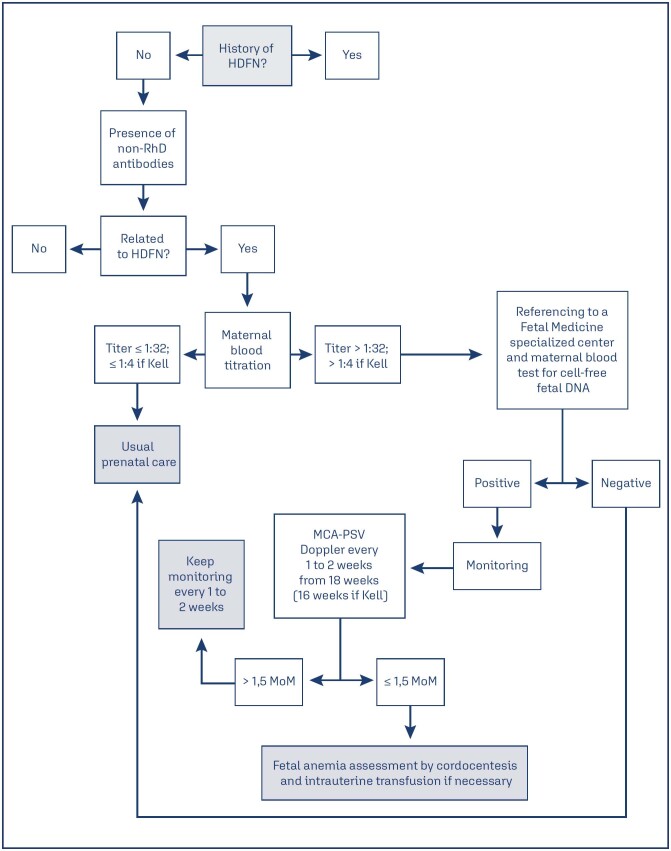
Flowchart of the screening and monitoring strategies in cases of non-RhD alloimmunization

The therapeutic options for those in whom disease is already installed and properly diagnosed do not differ much from the management of Rh alloimmunization. That is, during pregnancy, maternal therapies such as specific intravenous immunoglobulin, therapeutic plasma exchange and monoclonal antibodies can be used; fetal therapies such as intrauterine transfusion.^(1)^ Postnatal management, on the other hand, may involve transfusions in case of anemia and phototherapy or exchange transfusion in case of jaundice.^(1)^ Figure 1 shows the flowchart of the screening and monitoring strategies in cases of non-RhD alloimmunization.

## Conclusion

Alloimmunization in pregnancy is known to lead to HDFN, with possible catastrophic consequences for the fetus. The most important alloantigen is still RhD. However, as prevention methods are primarily aimed at anti-RhD antibodies, the others have been growing in proportion and clinical significance and therefore should be studied. Although there are several alloantigens capable of causing alloimmunization and their prevalence varies greatly from country to country, Rhc and Kell stand out as those with the highest risk and potential severity. However, recommendations for screening and management of alloimmunization caused by such antibodies are not widespread, so investigation often ends up being limited to Rh and ABO blood groups. Therefore, if other antibodies associated with risk of developing alloimmunization are detected, the above recommendations should be followed. The antibody titers in the maternal blood are evaluated and, if necessary, the production of the alloantigen by tests in the paternal blood, which, if positive, indicate the need for fetal DNA evaluation. Once the antigen production by the fetus is confirmed, periodic monitoring with Doppler of the MCA-PSV is recommended to evaluate the need for other procedures. Therefore, it is understood that non-RhD alloimmunization has global clinical importance and has well-described recommendations for its management.
